# Petits puits de la lèvre inférieure: pensez à la fistule labiale paramédiane

**DOI:** 10.11604/pamj.2014.19.203.5383

**Published:** 2014-10-24

**Authors:** Zakia Douhi, Fatima Zahra Mernissi

**Affiliations:** 1 Service de Dermatologie, Centre Hospitalier Universitaire Hassan II, Fès, Maroc

**Keywords:** Fistule, congénitale, lèvre inférieure, Fistula, congenital, lower lip

## Image en medicine

Nous rapportons le cas d'une fille de sept ans, sans antécédents particuliers, qui était vu en consultation de dermatologie pédiatrie pour deux lésions congénitales symétriques de la lèvre inférieure. Il n'y avait pas de cas similaires dans la famille. L'examen clinique trouvait deux lésions circulaires et symétriques par rapport à la ligne médiane de la lèvre inférieure, centrées par un petit pertuis millimétrique sans issu de liquide à la pression. La palpation ne trouvait pas de masse ni de formation kystique et l'examen endobuccal était sans particularités, notamment pas de fente palatine ou de fistules. Le diagnostic de fistules labiales paramédianes a était évoqué. La patiente a était adresser en chirurgie plastique pour excision. Les fistules labiales sont des petits puits congénitaux souvent asymptomatiques de la lèvre inférieure ou supérieure, paramédians ou bien commissuraux. Elles sont soit isolées soit associées à une fente labiale, labiopalatine ou palatine dans le cadre de syndrome de Van der Woude. C'est une rare anomalie héréditaire de transmission autosomique dominante avec une pénétrance incomplète et une expression variable inter et intrafamiliale. Les fistules se présentent sous la forme d'une dépression circulaire uni ou bilatérale à la surface du vermillon centrées par un pertuis, le plus souvent symétrique. Ces pertuis forment un canal délimité par la muqueuse labiale et qui dissèque le muscle orbiculaire suivant une profondeur variable de 1 à 25 mm. Habituellement, les fistules labiales inférieures sont asymptomatiques, néanmoins elles peuvent présenter un écoulement salivaire soit continu, soit intermittent. Des phénomènes inflammatoires secondaires à une inclusion alimentaire dans les pertuis peuvent être rencontrés. Aucun examen paraclinique n'est nécessaire. Le traitement est chirurgical, consiste en l'exérèse de tout le trajet fistuleux.

**Figure 1:**
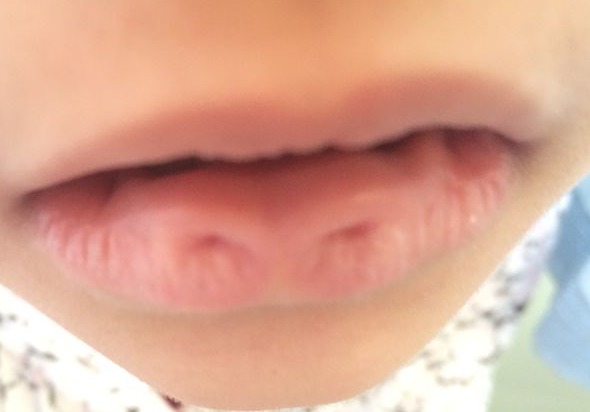
Fistule labiale inférieure paramédiane

